# Balanced excitation and inhibition in a spiking model of V1

**DOI:** 10.1186/1471-2202-14-S1-P184

**Published:** 2013-07-08

**Authors:** Filip Piekniewski, Micah Richert, Dimitry Fisher, Botond Szatmary, Csaba Petre, Sach Sokol, Eugene Izhikevich

**Affiliations:** 1 Brain Corporation, San Diego, CA 92121, USA; 2 Department of Neuroscience and The Zanvyl Krieger Mind/Brain Institute Johns Hopkins University, Baltimore, MD, USA

## Introduction

Experimental studies have shown that neuronal excitation is balanced with inhibition and spikes are triggered only when that fine balance is perturbed. It is also known that inhibition is critical for receptive field tuning, yet it is not clear what role is played by different types of inhibitory interneurons and how the corresponding balanced circuitry could emerge via spike timing dependent plasticity (STDP). To study these questions we have constructed a large-scale detailed spiking model of V1 involving a variety of simulated neurons: fast-spiking (FS) interneurons, low threshold spiking (LTS) interneurons and regular spiking (RS) neurons. We modeled layer 4 and layer 2/3 of the primary visual cortex and a number of projections between cell types in agreement with anatomical data.

Synaptic dynamics is governed by a set of STDP and activity dependent plasticity mechanisms for both inhibitory and excitatory synapses. The plasticity rules have been chosen to be in quantitative agreement with experiment where the data is available. For many of connections however, the data is either unavailable or noisy. In these cases plasticity rules were chosen based on a guided guess constrained by the requirement of structural stability of the system and expected response properties of cells to probing stimuli. Together, the plasticity rules lead to stable neuronal response and formation of orientation-selective receptive fields. The network learns simple and complex cells of a broad range of orientations and spatial frequencies.

The model converges to a balanced neurodynamics and biologically reasonable firing rates. Our study shows that in the presence of strong thalamic drive, plastic inhibition is necessary for feature selectivity. The FS cells remove DC component of the input while firing of the LTS cells imposes sparse response and balances out feedback excitation.

## Methods

**Figure 1:**
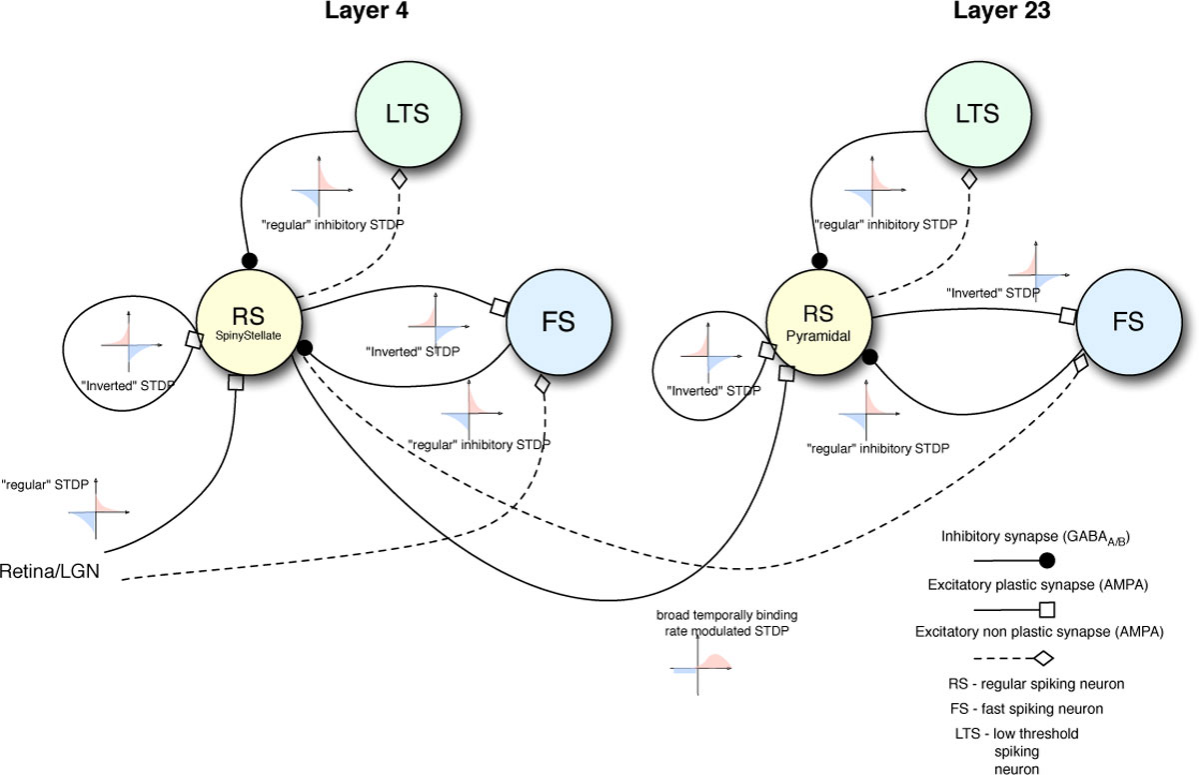
**The schematic representation of the connectivity**.

The model is built on a grid of single compartment phenomenological simple model [1] units with parameters set to resemble the firing pattern of regular spiking cells, fast spiking inhibitory interneurons and low threshold spiking neurons. The cells project within certain radii and the connections are controlled by spike timing dependent plasticity and activity dependent plasticity rules as schematically shown in Figure 1.

The input to the system is provided by a simulated spiking retina implementing the magnocellular pathway, where the stimulus strength is encoded with spike latency from the onset. The input to the system is a sequence of frames taken from a randomly generated saccade path over a natural image simulating a visual stimulus at 4-6 degrees eccentricity from the fovea.

## References

[B1] Izhikevich EM Simple model of spiking neurons IEEE Trans On Neural Networks 2003 14 6 1569 15 10.1109/TNN.2003.820440 18244602

